# Similarity-based future common neighbors model for link prediction in complex networks

**DOI:** 10.1038/s41598-018-35423-2

**Published:** 2018-11-19

**Authors:** Shibao Li, Junwei Huang, Zhigang Zhang, Jianhang Liu, Tingpei Huang, Haihua Chen

**Affiliations:** China University of Petroleum, College of Computer and Communication Engineering, Qingdao, Shandong 266580 China

## Abstract

Link prediction aims to predict the existence of unknown links via the network information. However, most similarity-based algorithms only utilize the current common neighbor information and cannot get high enough prediction accuracy in evolving networks. So this paper firstly defines the future common neighbors that can turn into the common neighbors in the future. To analyse whether the future common neighbors contribute to the current link prediction, we propose the similarity-based future common neighbors (SFCN) model for link prediction, which accurately locate all the future common neighbors besides the current common neighbors in networks and effectively measure their contributions. We also design and observe three MATLAB simulation experiments. The first experiment, which adjusts two parameter weights in the SFCN model, reveals that the future common neighbors make more contributions than the current common neighbors in complex networks. And two more experiments, which compares the SFCN model with eight algorithms in five networks, demonstrate that the SFCN model has higher accuracy and better performance robustness.

## Introduction

Many social, biological, and food-chain systems can be well described by networks, where nodes denote individuals, biological elements, and so on, and links represent the relations between nodes. The complex networks has therefore become a popular focus of many branches of science. An attractive research topic is link prediction, whose purpose is to predict the possibility or necessity of forming links between unconnected node pairs via the information of complex networks^1,2^. Thus Link prediction can predict the existing yet unknown links (the missing links) and the links that may appear in the future (the future links)^3,4^. With the amount of data increasing nowadays, Link prediction plays a more crucial role in recommendation system, data mining, complex networks, and so on. For instance, in the protein-protein interaction network of Yeast, 80% of the molecular interactions are still unknown. Whether a link between two nodes exists must be demonstrated by field and/or laboratorial experiments^5^. However, if the accurate prediction results are applied into the laboratorial experiments instead of blindly checking all possible interactions, the costs will be sharply reduced^6^. In the scientists cooperation network, link prediction helps to find the potential cooperation between scientists^7,8^. Besides, link prediction is also employed in recommending friends for online social networks^9,10^ and identifying spurious links in a noisy environment^11,12^.

Until now, many indexes for link prediction have been proposed. Generally, they are classified into three main models: models based on Markov chains^13–15^, models based on machine learning^16,17^ and models based on the similarity of topological structure^1,18^. Though the first two have high prediction accuracy in many networks, they don’t apply to the large-scale networks due to their high computational complexity. Nevertheless, models based on similarity can avoid such problems and easily obtain the information of networks. For instance, the Common Neighbor (CN) index, which is the most widely used index, just counts the number of common neighbors between node pair. Newman^19^ used this quantity in the study of collaboration networks, showing a positive correlation between the number of common neighbors and the probability that two scientists will collaborate in the future. By taking into account the common neighbors number and the degrees of two nodes, Salton et al. pointed out the Salton index^20^; Leicht, Holme and Newman proposed the LHN index^21^. To characterize the topological similarity between reactants in the metabolic network, Ravasz E. and Somera A. L. et al. proposed the hub promoted index (HPI)^22^. HPI index insists that the links adjacent to hubs are likely to be assigned high scores since the denominator is determined by the common neighbors number and the lower degree. To measure with the opposite effect of hubs, Zhou and Lü et al. put forward the hub depressed index (HDI)^23^. Furthermore, they proposed the resource allocation index (RA)^23^. Motivated by the resource allocation dynamics on complex networks, the RA index can effectively improve the accuracy by restraining the contributions of large-degree common neighbors. Additionally, Liu et al. proposed a Local Naive Bayes (LNB) model^24^, which insists that different common neighbors play different roles and make different contributions. Based on the LNB model, they improved the CN, RA and AA index. As the similarity indexes can predict links in networks, we can apply the similarity indexes to evaluate the evolving mechanisms for the evolving networks^1^.

Obviously, similarity-based algorithms for link prediction can predict the future links by using the current common neighbors information^25^. However, on above principles of the similarity indexes, some nodes, currently not common neighbors, can turn into the common neighbors in the future. More importantly, these nodes raise a series of new questions worth exploring. First of all, it is whether these nodes contribute to the current prediction between node pair. Although the previous algorithms have proved that the current common neighbors can promote two nodes to connect, people still doubt whether nodes, which are currently not a common neighbor but can become a common neighbor in the future, are also helpful in the current link prediction. Second, if they do make contribution, then how we can locate these nodes and measure their contribution, simultaneously. Previous algorithms can easily count the number of the current common neighbors by only analyzing the network topology. However, the nodes described above have not yet become the common neighbors, and they can get different topology structures when they are together with the node pairs and their surrounding nodes. These lead to the challenge of locating these nodes and measuring their contributions via a simple method.

To address the above problems clearly, firstly, we define nodes, which are currently not common neighbors but can turn into the common neighbors in the future, as the future common neighbors and divide them into three types according to their topology structure with other nodes. Second, we propose the similarity-based future common neighbors (SFCN) model for link prediction. The SFCN model accurately finds out all the future common neighbors, besides the current common neighbors. And simultaneously, it can also measure their contributions by only using the existing similarity indexes. We also design and observe three MATLAB simulation experiments. First, we conduct a priori experiment on *α* and *β* in FWFB network. The results provide strong evidence that the future common neighbors have more positive contribution than the common neighbors in complex networks. Second, by comparing the SFCN model with eight similarity-based algorithms in five networks, we find that the SFCN model has higher prediction accuracy from the whole perspective. Third, the experiments, where we change the ratio of the training set to the probe set in five networks, also demonstrates that the SFCN model has better performance robustness. So, the proposed SFCN model has higher accuracy and performance robustness than popular algorithms, and the future common neighbors is necessary to be considered for link prediction in evolving networks.

## Results

### Network and problem description

A network can be represented by an undirected network *G*(*V*, *E*) without self-connections and multiple links between node pair. In *G*(*V*, *E*), *V* is the set of nodes, and *E* is the set of links. Then |*V*| represents the quantity of nodes in *V*. Define the fully connected network as *U* that contains (|*V*|(|*V*| − 1))/2 links. So, *U-E* is the set of the nonexistent links. To evaluate the prediction accuracy of algorithms, we divide the observed link set *E* into the training set *E*^*T*^ and the probe set *E*^*P*^ randomly. *E*^*T*^ is the known information while *E*^*P*^ is the unknown information. Obviously, *E* = *E*^*P*^ ∪ *E*^*T*^, and *ϕ* = *E*^*P*^ ∩ *E*^*T*^. Accurately detecting the missing links or the future links from *U-E* is the purpose of link prediction. Give the link between node pair (*x*, *y*) in *U* a score (*s*_*x*,*y*_), which is calculated by the link prediction algorithm. All the nonexistent links are sorted in descending order according to their scores, and the links at the top are most likely to exist.

### The future common neighbors

Most similarity-based algorithms for link prediction predict the future links by using the current common neighbors. However, on the prediction principles of the above similarity indexes, some nodes, which are currently not common neighbors, can turn into the common neighbors in the future. To analyze whether such nodes are factors that contribute to the current prediction between node pair, and in order to accurately locate these nodes to measure their contribution, we define them as the future common neighbors and propose the similarity-based future common neighbors model for link prediction in evolving networks.

**Figure 1:**
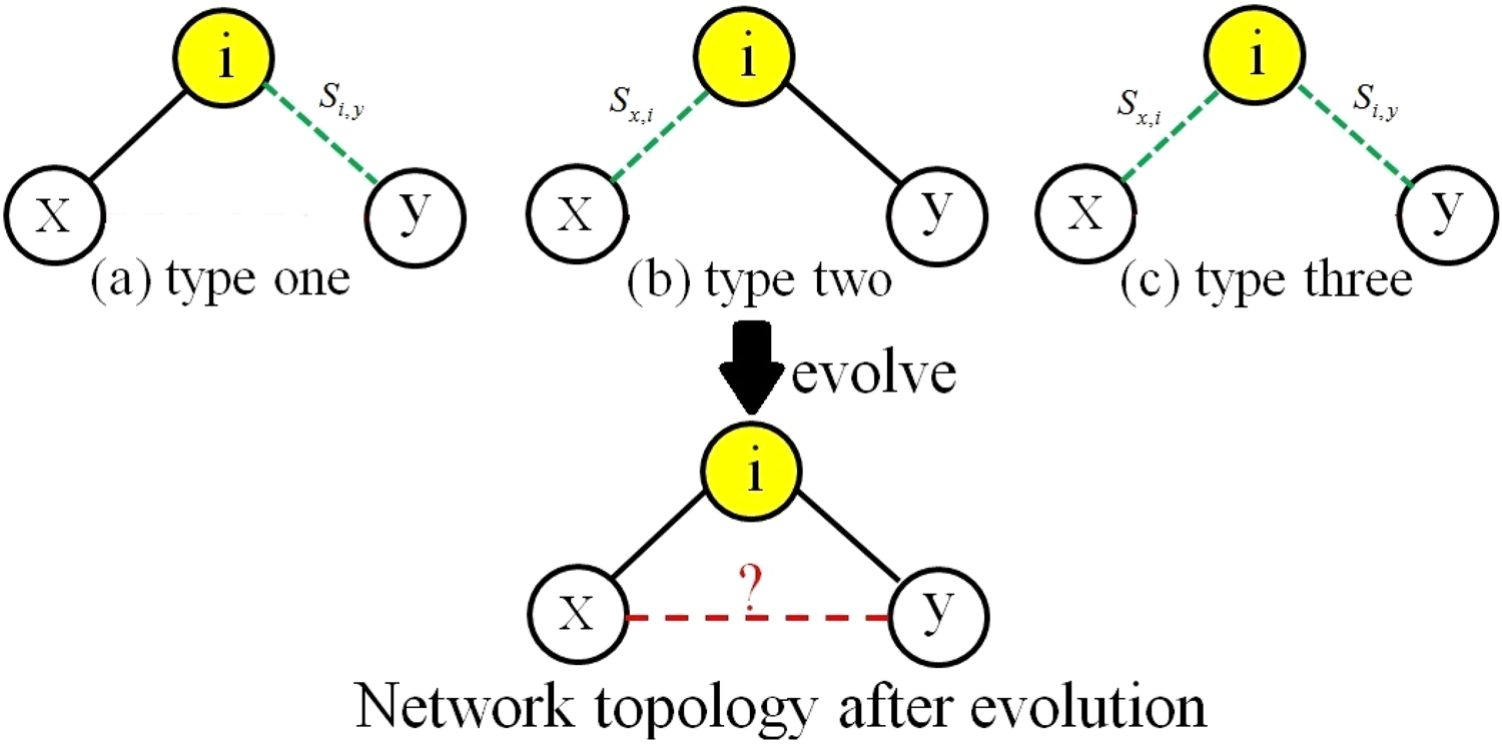
Three types of the future common neighbors.

The future common neighbors are nodes that are currently not the common neighbors but can turn into the common neighbors in the future on the principle of the similarity index. According to their topology with other nodes, the future common neighbors are divided into three types shown in Fig. 1, where *x* and *y* are the target node pair for link prediction. The first future common neighbor, like node *i* in Fig. 1(a), has a direct link with *x* while no direct link with *y*. Currently, the similarity score between *i* and *y* is *s*_*i*,*y*_. According to the prediction principle of the similarity algorithm, *i* and *y* may form a link in the future (the greater the *s*_*i*,*y*_, the greater the probability of forming a link). Therefore, *i* has connected with *y* and turn into the common neighbor between *x* and *y* in a future time. The second future common neighbor, like *i* in Fig. 1(b), has direct link with *y* while no direct link with *x*. The third future common neighbors does not connect with both *x* and *y*, seen node *i* in Fig. 1(c). According to the existing similarity indexes for link prediction, if *s*_*x*,*i*_ and *s*_*i*,*y*_ are great enough, *i* are will form links with both *x* and *y*. Thus, the *i* in Fig. 1(c) are also a common neighbor between *x* and *y* in a future time.

### Similarity-based future common neighbors model

Combining the future common neighbors topology with the similarity-based indexes, this paper designs the similarity-based future common neighbors model. The model is to accurately find out all the future common neighbors in complex networks and effectively measure their contributions.

**Figure 2:**
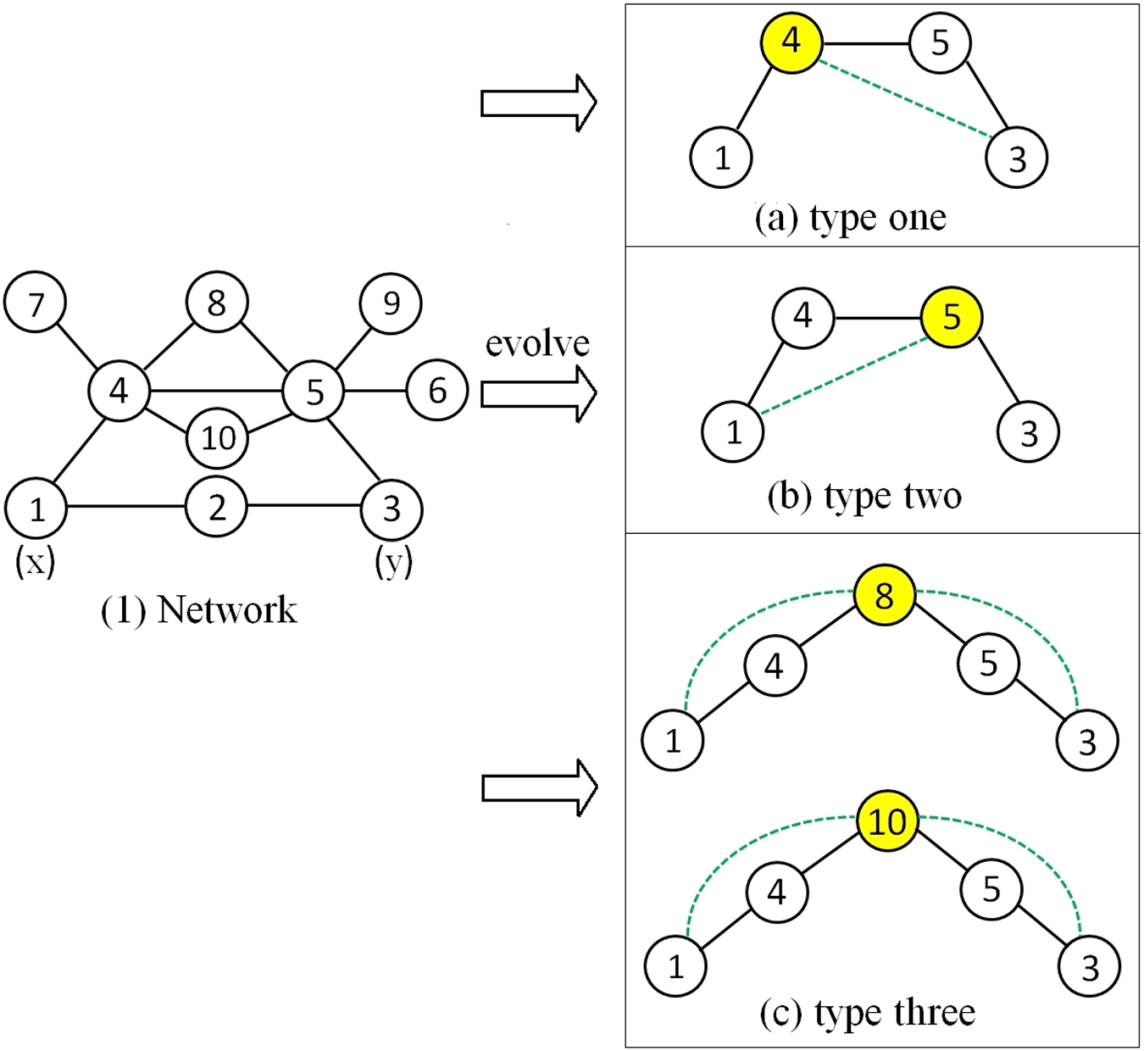
The process of identifying the future common neighbors from the chaotic network. The yellow nodes are the future common neighbors between *x* and *y*.

Taking the chaotic network in Fig. 2 as an example, for node *i* (*i* = 1, 2, 3, …, |*V*|), we assume that ${s}_{x,i}^{C2}$ is a similarity score between *x* and *i*, and ${s}_{x,i}^{C2}$ is calculated by any classical similarity-based algorithms that we mark as *C*2. Therefore, ${s}_{x,i}^{C2}$ also symbolizes the possibility of forming a link between *x* and *i* on the principle of *C*2 when the network is evolving. We make *r*_*i*,*y*_ indicate whether *i* and *y* are connected (*r*_*i*,*y*_ = 1 if *i* and *y* are connected, otherwise *r*_*i*,*y*_ = 0), which can be obtained from the observed networks. Similarly, ${s}_{i,y}^{C2}$ is a similarity score between *i* and *y* calculated by algorithms *C*2; *r*_*x*,*i*_ represents whether *x* and *i* are connected.

The SFCN model identifies the above three types of the future common neighbors from chaotic network by employing their topological rules. (1) *i* is the first type of the future common neighbor only when ${s}_{x,i}^{C2}\cdot {r}_{i,y}\ne 0$. It is necessary to note that we set ${s}_{x,i}^{C2}=0$ and *r*_*i*,*y*_ = 0 when *i* = *x* or *i* = *y* in order to keep non self-connections. (2) The rest rules can be deduced by analogy, *i* is the second type of the future common neighbor if ${r}_{x,i}\cdot {s}_{i,y}^{C2}\ne 0$. (3) And *i* is the third type of the future common neighbor if and only if ${s}_{x,i}^{C2}\cdot {s}_{i,y}^{C2}\ne 0$.

To accumulate the contributions of the future common neighbors, which meet the above rules, we construct four vectors for *x* and *y* in eqs 1, 2, 3, 4.

1 $$\begin{array}{l}{{\boldsymbol{\Gamma }}}_{{\bf{x}}}=({r}_{x\mathrm{,1}}\,\cdots \,{r}_{x,x-1}\,0\,{r}_{x,x+1}\,\cdots \,0),\end{array}$$

2 $$\begin{array}{c}{({{\boldsymbol{\Gamma }}}_{{\bf{y}}})}^{T}={({r}_{1,y}\cdots {r}_{y-1,y}0{r}_{y+1,y}\cdots 0)}^{T},\end{array}$$

3 $${{\bf{S}}}_{{\bf{x}}}^{{\bf{C}}{\bf{2}}}=({s}_{x\mathrm{,1}}^{C2}\,\cdots \,{s}_{x,x-1}^{C2}\,0\,{s}_{x,x+1}^{C2}\,\cdots \,0),$$

4 $$\begin{array}{c}{({{\bf{S}}}_{{\bf{y}}}^{{\bf{C}}{\bf{2}}})}^{T}={({s}_{1,y}^{C2}\cdots {s}_{y-1,y}^{C2}0{s}_{y+1,y}^{C2}\cdots 0)}^{T},\end{array}$$

where the superscript ^*T*^ denotes matrix transposition, and the black highlighted parts are the row vectors or column vectors. **Γ**_***x***_ stores the connections of *x* to all nodes. ${{\bf{S}}}_{{\bf{x}}}^{{\bf{C}}{\bf{2}}}$ stores the similarity scores of *x* to all nodes. Similarly, (**Γ**_***y***_)^*T*^ stores the connections of *y* to all nodes. And ${({{\bf{S}}}_{{\bf{y}}}^{{\bf{C}}{\bf{2}}})}^{T}$ stores the similarity scores between *y* and all other nodes.

Therefore, we get the similarity-based future common neighbors model as eq. 5:

5 $$\begin{array}{rcl}{s}_{x,y}^{SFCN} & = & \alpha \cdot {s}_{x,y}^{C1}+\beta \cdot ({{\boldsymbol{\Gamma }}}_{{\bf{x}}}\cdot {({{\bf{S}}}_{{\bf{y}}}^{{\bf{C}}{\bf{2}}})}^{T}+{{\bf{S}}}_{{\bf{x}}}^{{\bf{C}}{\bf{2}}}\cdot {({{\boldsymbol{\Gamma }}}_{{\bf{y}}})}^{T}+{{\bf{S}}}_{{\bf{x}}}^{{\bf{C}}{\bf{2}}}\cdot {({{\bf{S}}}_{{\bf{y}}}^{{\bf{C}}{\bf{2}}})}^{T})\\  & = & \alpha \cdot {s}_{x,y}^{C1}+\beta \cdot \sum _{i=1}^{|V|}({r}_{x,i}\cdot {s}_{i,y}^{C2}+{s}_{x,i}^{C2}\cdot {r}_{i,y}+{s}_{x,i}^{C2}\cdot {s}_{i,y}^{C2}),\end{array}$$

where ${s}_{x,y}^{C1}$ is the similarity score between *x* and *y*, and ${s}_{x,y}^{C1}$ is calculated by any similarity algorithm that we temporarily mark as *C*1. C1 and C2 are two similarity algorithms, and they can be the same or different. The two free parameters, *α* and *β*, is to adjust the contributions of the current common neighbors and the future common neighbors, respectively. When *α* ≠ 0 and *β* = 0, the model only considers the current common neighbors contributions. When *α* = 0 and *β* ≠ 0, the model only considers the future common neighbors contributions. Both C1 and C2 are the. A special case, when both *C*1 and *C*2 are represented by CN algorithm and only the first class of the future common neighbors are considered, the model degenerates into the LP index. In a word, the SFCN model, employed in evolving networks, takes into account the contributions of the future common neighbors besides the current common neighbors.

### Example 1

This section gives an example of how to find the future common neighbors between node pair and how to measure the contributions of three future common neighbors. Suppose *C*1 and *C*2 are the LHN and RA algorithms, respectively. Take the network in Fig. 2 as an example and treat nodes (1, 3) as the target node pair (*x*, *y*). Then we can get four vectors:

6 $$\begin{array}{l}{{\boldsymbol{\Gamma }}}_{{\bf{1}}}=(0\,1\,0\,1\,0\,0\,0\,0\,0\,0),\end{array}$$

7 $$\begin{array}{c}{({{\boldsymbol{\Gamma }}}_{3})}^{T}={(0100100000)}^{T},\end{array}$$

8 $$\begin{array}{l}{{\bf{S}}}_{{\bf{1}}}^{{\bf{C}}{\bf{2}}}=(0\,0\,0\,0\,\frac{1}{5}\,0\,\frac{1}{5}\,\frac{1}{5}\,0\,\frac{1}{5}),\end{array}$$

9 $${({{\bf{S}}}_{{\bf{3}}}^{{\bf{C}}{\bf{2}}})}^{T}={(000\frac{1}{6}0\frac{1}{6}0\frac{1}{6}\frac{1}{6}\frac{1}{6})}^{T}.$$

From the calculation process (eq. 10), it is easy to observe that only node 4 is the first type of the future common neighbors. And the contribution of node 4 to (1, 3) is $\frac{1}{6}\cdot \beta$.

10 $$\begin{array}{l}{{\boldsymbol{\Gamma }}}_{{\bf{1}}}\cdot {({{\bf{S}}}_{{\bf{3}}}^{{\bf{C}}{\bf{2}}})}^{T}=\sum _{i=1}^{|V|}\,({r}_{\mathrm{1,}i}\cdot {s}_{i\mathrm{,3}}^{C2})={r}_{\mathrm{1,4}}\cdot {s}_{\mathrm{4,3}}^{C2}=\frac{1}{6}\mathrm{.}\end{array}$$

We can also observe that only node 5 is the second type of the future common neighbors from the eq. 11:

11 $$\begin{array}{c}{{\bf{S}}}_{{\bf{1}}}^{{\bf{C}}{\bf{2}}}\cdot {({{\boldsymbol{\Gamma }}}_{{\bf{3}}})}^{T}=\sum _{i=1}^{|V|}\,({s}_{1,i}^{C2}\cdot {r}_{i,3})={r}_{1,5}\cdot {s}_{5,3}^{C2}=\frac{1}{5}.\end{array}$$

At last, we can check out that 8 and 10 are the third type of the future common neighbors through eq. 12:

12 $$\begin{array}{c}{{\bf{S}}}_{{\bf{1}}}^{{\bf{C}}{\bf{2}}}\cdot {({{\bf{S}}}_{{\bf{3}}}^{{\bf{C}}{\bf{2}}})}^{T}=\sum _{i=1}^{|V|}\,({s}_{1,i}^{C2}\cdot {s}_{i,3}^{C2})={s}_{1,8}^{C2}\cdot {s}_{8,3}^{C2}+{s}_{1,10}^{C2}\cdot {s}_{10,3}^{C2}=\frac{1}{15}.\end{array}$$

Therefore, the contribution of the future common neighbors is $\beta \cdot (\frac{1}{6}+\frac{1}{5}+\frac{1}{15})=\frac{13}{30}\cdot \beta$.

### Evaluation Metrics

In the Experiments, we introduce two standard metrics to quantify the prediction accuracy: the *AUC*^26^ (area under the receiver operating characteristic curve) and *precision*^27^. The AUC evaluate the algorithms performance according to the whole list. The AUC is comprehended as the probability that a link randomly chosen from set *E*^*T*^ has a much higher score than a link randomly chosen from nonexistent link *U-E*. In the *n* times independent comparisons, we select a link from *E*^*T*^ and *U-E* respectively. Define their similarity scores as *S*1 and *S2*. When *S*1 > *S2*, set *n*′ = *n*′ + *1*; when *S1*  = *S2*, set *n*″ = *n*″ + *1* (*n*′ and *n*″ are initialed as 0, *n* = *n*′ + *n*″). So, the *AUC* can be defined as eq. 13:

13 $$\begin{array}{l}{\rm{AUC}}=(n^{\prime} +0.5n^{\prime\prime} )/n\mathrm{.}\end{array}$$

Different from the AUC, precision focuses on the links with top ranks or highest scores. It is the ratio of correct links recovered out of the top *L* links in the candidate list generated by each link predictor. Assume *L*_*r*_ links are accurately predicted among the top-*L* links. Then the precision can be defined as eq. 14:

14 $${\rm{Precision}}=\frac{{L}_{r}}{L}\mathrm{.}$$

### Datasets of real networks

In order to compare the prediction accuracy of the SFCN model with the eight mainstream indexes mentioned in this paper, we do MATLAB simulation experiments in five real networks: the network of scientific communication (NS)^28^, the US political blogs network (PB)^29^, the protein interaction network (Yeast)^30^, the neural network of C.elegans (CE)^31^, the food web network of florida bay (FWFB)^32^. All datasets of the five networks can be seen in the electronic supplementary material. The basic features of those networks are summarized in Table 1.

**Table 1:** Details of networks.

Networks	|*V*|	|*E*|	<*d*>	<*k*>	<*H*>	<*C*>	<*r*>
CE	297	2148	2.46	14.4646	1.8008	0.3079	−0.163
FWFB	128	2075	1.78	32.4219	1.2370	0.3346	−0.112
NS	379	914	4.93	4.82	1.66	0.798	−0.082
PB	1222	16714	2.74	27.3552	2.9707	0.3600	−0.221
Yeast	2375	11693	5.09	9.8467	3.4756	0.3883	0.454

|*V*| and |*E*| are the number of nodes and links, respectively. <*d*> is the average shortest distance between node pairs. <*k*> is the average degree, and <*H*> denotes the degree heterogeneity. <*C*> represents the clustering coefficient. <*r*> is the assortative coefficient.

The metrics that characterize the networks can be seen in the caption of Table 1. We find that NS, PB and CE have similar characteristics, including the high clustering coefficient. Nevertheless, for FWFB network, the relation between predator and prey makes the network have a larger average degree and a shorter average distance between node pair.

### Existing similarity indexes based on topological structure

Here, we introduce eight mainstream similarity indexes to compare with the SFCN model.

- CN. Let Γ_*x*_ be the set of neighbors of *x*. The CN index proposes that node pair (*x*, *y*) are more likely to connect if they have more common neighbors, namely:
  15 $${s}_{x,y}^{CN}=|{{\rm{\Gamma }}}_{x}\cap {{\rm{\Gamma }}}_{y}|\mathrm{.}$$
- Salton^20^. It is defined as:
  16 $${s}_{x,y}^{Salton}=\frac{|{{\rm{\Gamma }}}_{x}\cap {{\rm{\Gamma }}}_{y}|}{\sqrt{{k}_{x}{k}_{y}}},$$
  where *k*_*x*_ is the degree of node *x*.
- RA^23^. The RA index assumes each transmitter has a unit of resource and will equally distributed to all its neighbors, concluded as:
  17 $${s}_{x,y}^{RA}={\sum }_{z\in {{\rm{\Gamma }}}_{x}\cap {{\rm{\Gamma }}}_{y}}\,\frac{1}{{k}_{z}}.$$
- HPI^22^. It is defined as:
  18 $${s}_{x,y}^{HPI}=\frac{|{{\rm{\Gamma }}}_{x}\cap {{\rm{\Gamma }}}_{y}|}{{\rm{\min }}\,\{{k}_{x},{k}_{y}\}}\mathrm{.}$$
- HDI^23^. It is defined as:
  19 $${s}_{x,y}^{HDI}=\frac{|{{\rm{\Gamma }}}_{x}\cap {{\rm{\Gamma }}}_{y}|}{{\rm{\max }}\,\{{k}_{x},{k}_{y}\}}\mathrm{.}$$
- Leicht-Holme-Newman index (LHN)^21^. The LHN index is defined as:
  20 $${s}_{x,y}^{LHN}=\frac{|{{\rm{\Gamma }}}_{x}\cap {{\rm{\Gamma }}}_{y}|}{{k}_{x}{k}_{y}}\mathrm{.}$$
- LNBRA^24^. The LNBRA index is an improvement in RA index based on the LNB model, defined as:
  21 $${s}_{x,y}^{LNBRA}=\sum _{z\in {{\rm{\Gamma }}}_{x}\cap {{\rm{\Gamma }}}_{y}}\,\frac{1}{{k}_{z}}({\mathrm{log}}_{2}\eta +{\mathrm{log}}_{2}{R}_{z}),$$
  where *η* and R _*z*_ are defined as:
  22 $$\eta =\frac{|V|(|V|-1)}{2|{E}^{T}|}-1,$$
  23 $${R}_{z}=\frac{{N}_{{\rm{\Delta }}z}+1}{{N}_{\nabla z}+1},$$
  where *N*_Δ*z*_ and *N*_▽*z*_ are respectively the numbers of connected and disconnected node pairs which have a common neighbor *z*.
- Local Path (LP)^33^. This index considers the number of different orders, defined as:

24 $${s}_{x,y}^{LP}={({A}^{2})}_{x,y}+\alpha \cdot {({A}^{3})}_{x,y},$$

where *α* is an adjustable parameter and *A* is the adjacency matrix of network. (*A*^*i*^)_*x*,*y*_ represents the quantity that the order length is equal to *i* between *x* and *y*.

### Experiments and performance analysis

In this section, we do three experiments and make corresponding analysis for three purposes. In the first and second experiments, the *E*^*T*^ contains 90% of links, while the remaining 10% of links constitute the *E*^*P*^. In addition, all the following results are returned with the average over 100 independent experiments.

**Figure 3:**
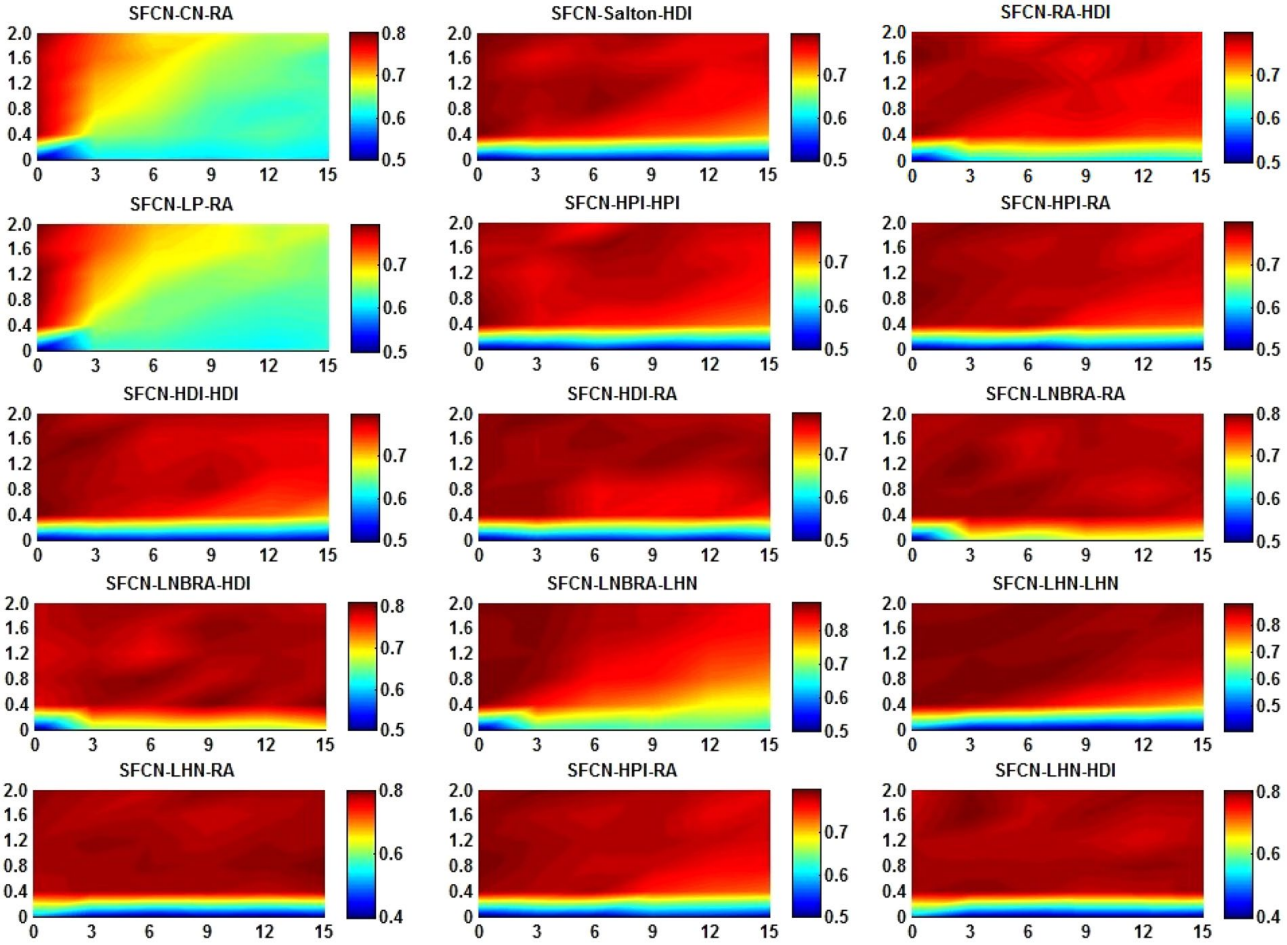
AUC sensitivity analysis of the SFCN model in FWFB network. X-axis is the *α* value that is taken from 0 to 15 at intervals of 3. Y-axis is the *β* value that is taken from 0 to 2 at intervals of 0.4.

For the first experiment, to verify whether the contribution of the future common neighbors is necessary, we conducted priori experiments on *α* and *β* in FWFB network. Step 1, since the training set (*E*^*T*^) is known, we divide *E*^*T*^ into the sub-training set (*E*^*T*1^) and the sub-probe set (*E*^*P*1^) to learn the values of *α* and *β* in step 2. Step 2, we apply the SFCN model to the sub-training set in order to obtain the similarity scores of the sub-probe set and get the AUC that varies with *α* and *β*. In this way, it is easy to select the numerical values of *α* and *β* with high AUC for the SFCN model. The experimental results are shown in Fig. 3. Before that, it is necessary to consider the two limit problems. When *α* = 0, the current common neighbors in the SFCN model do not make any contribution, and only the future common neighbors make contributions. When *β* = 0, there are only contribution from the current common neighbors, and the future common neighbors do not make any contributions for link prediction. We can get two results from the Fig. 3. First, the AUC when *β* = 0 is much lower than that when *β*≠0. Second, the SFCN model can obtain highest AUC when *α* and *β* are adjust to a suitable value. For example, for the SFCN-CN-RA, SFCN-Salton-HDI, and SFCN-LNBRA-LHN algorithms, we should set *α* smaller and *β* larger to get a higher prediction accuracy in FWFB network. These two results illustrate the important contribution of the future common neighbors.

Therefore, in the second and the third experiments later, we set *α* = 9 and *β* = 1, which meet the above condition.

The second experiment is to compare the SFCN model with other eight similarity-based indexes, including the CN, HDI, HPI, LP, RA, Salton, LNBRA and LHN index. The prediction results of AUC and precision are listed in Tables 2 and 3 for details, respectively. Most comparative experiments, in the Table 2, clearly demonstrate that the SFCN model has the best or close to the best AUC, especially in the FWFB and Yeast networks. Taking the FWFB network as an example for analysis, we can see that there are 2075 links but only 128 nodes from the Table 1. And the average degree is as high as 32.422 while the average aggregation coefficient is low to 0.3346, which indicate that there are many random connections and high obscure similarity between the clusters in the FWFB network. These are the reasons why all nodes in the FWFB network have the tendency to gather and form some unknown clusters with the network evolving. The SFCN model takes into account the network evolution tendency via the principle of similarity index. In detail, the model has greatly improved the AUC in FWFB network by regarding the future common neighbors as the evolution direction. Moreover, Table 3 demonstrates that 90% of the precision results, predicted based on the SFCN model, are equal to or higher than that predicted based their original algorithm. For example, the precision results of the SFCN-HDI-RA algorithm are much higher than those of the original HDI algorithm in most network, because the contributions of the future common neighbors are taken into account.

**Table 2:** There are the prediction accuracy results, measured by AUC, of classic indexes and corresponding algorithms based on the SFCN model in five real networks.

AUC	CE	FWFB	NS	PB	Yeast
CN	0.846	0.616	0.989	0.925	0.917
SFCN-CN-RA	0.876	0.659	0.989	0.939	0.971
Salton	0.802	0.532	0.984	0.880	0.914
SFCN-Salton-HDI	0.851	0.793	0.984	0.937	0.974
RA	0.871	0.598	0.977	0.927	0.924
SFCN-RA-HDI	0.872	0.794	0.991	0.938	0.975
LP	0.861	0.633	0.980	0.938	0.971
SFCN-LP-RA	0.872	0.666	0.990	0.942	0.978
HPI	0.804	0.528	0.979	0.855	0.912
SFCN-HPI-HPI	0.811	0.762	0.985	0.901	0.972
SFCN-HPI-RA	0.865	0.790	0.989	0.945	0.974
HDI	0.775	0.527	0.980	0.873	0.914
SFCN-HDI-HDI	0.849	0.782	0.985	0.935	0.976
SFCN-HDI-RA	0.889	0.795	0.990	0.947	0.977
LNBRA	0.863	0.659	0.980	0.928	0.920
SFCN-LNBRA-RA	0.883	0.796	0.993	0.949	0.977
SFCN-LNBRA-HDI	0.881	0.809	0.992	0.942	0.975
SFCN-LNBRA-LHN	0.878	0.843	0.989	0.941	0.975
LHN	0.725	0.390	0.974	0.766	0.906
SFCN-LHN-LHN	0.810	0.891	0.983	0.891	0.974
SFCN-LHN-RA	0.876	0.797	0.992	0.947	0.977
SFCN-LHN-LP	0.806	0.704	0.974	0.928	0.961
SFCN-LHN-HDI	0.839	0.798	0.984	0.936	0.976

**Table 3:** There are the prediction accuracy results, measured by precision (top-100), of classic indexes and corresponding algorithms based on the SFCN model in five real networks.

Precision	CE	FWFB	NS	PB	Yeast
CN	0.198	0.094	0.396	0.460	0.678
SFCN-CN-RA	0.202	0.108	0.404	0.464	0.766
Salton	0.012	0.008	0.290	0.000	0.024
SFCN-Salton-HDI	0.012	0.010	0.260	0.000	0.032
RA	0.124	0.094	0.564	0.256	0.520
SFCN-RA-HDI	0.130	0.098	0.566	0.278	0.440
LP	0.124	0.112	0.312	0.400	0.654
SFCN-LP-RA	0.140	0.112	0.324	0.410	0.696
HPI	0.026	0.068	0.556	0.224	0.860
SFCN-HPI-HPI	0.036	0.388	0.192	0.224	0.868
SFCN-HPI-RA	0.116	0.382	0.162	0.566	0.896
HDI	0.032	0.008	0.310	0.002	0.030
SFCN-HDI-HDI	0.086	0.360	0.320	0.516	0.900
SFCN-HDI-RA	0.106	0.360	0.294	0.590	0.882
LNBRA	0.131	0.162	0.544	0.252	0.586
SFCN-LNBRA-RA	0.136	0.166	0.554	0.250	0.580
SFCN-LNBRA-HDI	0.130	0.164	0.564	0.278	0.602
SFCN-LNBRA-LHN	0.132	0.154	0.580	0.260	0.586
LHN	0.000	0.014	0.138	0.000	0.010
SFCN-LHN-LHN	0.000	0.026	0.138	0.000	0.014
SFCN-LHN-RA	0.000	0.014	0.138	0.000	0.012
SFCN-LHN-LP	0.000	0.020	0.138	0.000	0.012
SFCN-LHN-HD	0.000	0.018	0.140	0.000	0.010

**Figure 4:**
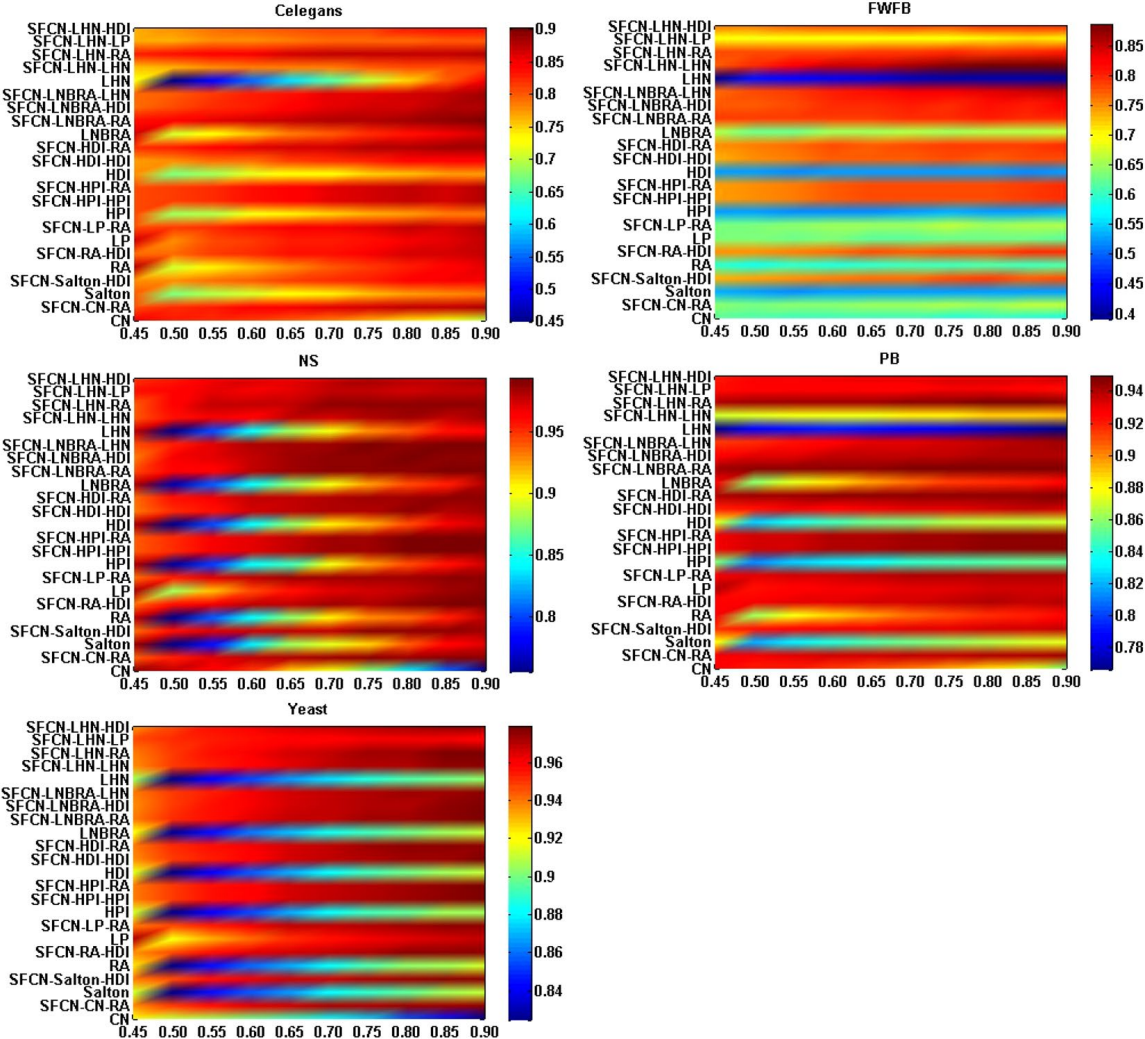
The AUC of different algorithms with different ratio of training sets to probe sets in real networks. X-axis is the ratio, and Y-axis is the each algorithm.

Finally, in order to explore the robustness, we change the ratio of training set to probe set in the third experiment. The lower the ratio, the more links information that should be predicted^34^. That is to say, there are less number of the known connected links and more number of the unknown links when the ratio is small. It is obviously to obtain two results from the Fig. 4. On the one hand, when the ratio is the same, the algorithms based on SFCN model have higher prediction accuracy results (measured by AUC) than their corresponding original algorithms. For instance, the SFCN-LHN-RA, SFCN-LHN-LP and SFCN-LHN-HDI algorithms have higher AUC compared with the original LHN algorithms when the ratio is the same. On the other hand, even when the ratio is low, the algorithms based on SFCN model still get high AUC, which indicates that the SFCN model has higher stability. Therefore, the SFCN model has better performance in prediction accuracy and stability even when there is few links information.

## Discussion

Exploring what factors can provide a positive impact on link prediction is an important and challenging problem. In this paper, we firstly discover the existence of the future common neighbors, which are classified into three types according to their topological structure with other nodes. Then, to investigate whether the future common neighbors can make positive contribution for current link prediction, we propose the similarity-based future common neighbors model (SFCN), which accurately locates all the future common neighbors and effectively measure their contributions in complex networks, besides the current common neighbors.

We design three simulation experiments via the MATLAB for three different purposes. First, we conduct priori experiments on *α* and *β* in FWFB network. The results provide strong evidence that the future common neighbors can make great contribution than current common neighbors in complex networks. In the second experiments, we compare the SFCN model with eight algorithms in five networks, finding that the SFCN model has higher prediction accuracy, especially the AUC in the FWFB and Yeast networks. Third, in order to verify whether the SFCN model can get great accuracy when the known link information is little, we change the ratio of the training set to the probe set in five networks. And the experiment results show that the SFCN model has better performance robustness, even when the ratio is low to 0.45, compared with eight similarity-based algorithms. Therefore, the proposed SFCN model has higher accuracy and performance robustness than popular similarity-based algorithms, and the future common neighbors make more positive contribution than the current common neighbors that is widely used nowadays.

Some extensions of this work deserve further exploration. One is that we are limited to the current common neighbors and the future common neighbors in evolving networks. It is meaningful to research the contribution of the future nodes and the future links. For example, current path-based algorithms only consider the contribution of the existing paths currently, so it is significative to further exploit whether and how much the future paths, which are not existing currently but will exist after once prediction, can make a positive impact on current link prediction.

## Methods

### Algorithm of the SFCN model for link prediction

The adjacency matrix *E* is a sparse matrix of the complex network. And the pseudocode of the SFCN model is presented in algorithm 1.

**Algorithm 1:**
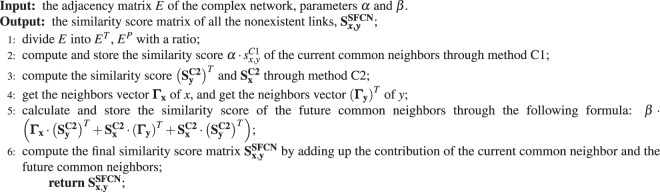
Algorithm of the proposed SFCN framework.

### Complexity analysis

This part give a simple complexity analysis of the proposed SFCN model. The most time-consuming part occurs in computing the contribution of the future common neighbors. The time cost of (${{\boldsymbol{S}}}_{{\boldsymbol{y}}}^{{\boldsymbol{C}}{\bf{2}}}$) is *O*(|*V*||*V*|), and the time cost of (${{\boldsymbol{S}}}_{{\boldsymbol{x}}}^{{\boldsymbol{C}}{\bf{2}}}$) is *O*(|*V*||*V*|). Thus the total time cost of the future common neighbors is 3⋅*O*(|*V*||*V*||*V*|). Since complex network can be simplified as an sparse matrix, the final computational complexity is much less than 3⋅*O*(|*V*||*V*||*V*|).

## Electronic supplementary material

Data sets of five networks.zip
